# Anti-*Enterococcus Faecalis*, Cytotoxicity, Phytotoxicity, and Anticancer Studies on *Clausena excavata* Burum. f. (Rutaceae) Leaves

**DOI:** 10.1155/2021/3123476

**Published:** 2021-02-23

**Authors:** Shaymaa Fadhel Abbas Albaayit, Rukesh Maharjan, Rasedee Abdullah, Mohd Hezmee Mohd Noor

**Affiliations:** ^1^Department of Biology, College of Science, University of Baghdad, Baghdad, Iraq; ^2^H.E.J. Research Institute of Chemistry, International Center for Chemical and Biological Sciences, University of Karachi, Karachi, Pakistan; ^3^Department of Veterinary Laboratory Diagnosis, Faculty of Veterinary Medicine, Universiti Putra Malaysia, 43400 Serdang, Selangor, Malaysia; ^4^Department of Veterinary Preclinical Sciences, Faculty of Veterinary Medicine, Universiti Putra Malaysia, 43400 Serdang, Selangor, Malaysia

## Abstract

**Background:**

*Clausena excavata* Burum. f. has long been applied in ethnomedicine for the treatment of various disorders like rhinitis, headache, cough, wound healing, fever, and detoxification. This study is aimed at investigating the antibacterial activity against *Enterococcus faecalis* ATCC 49532 using AlamarBlue assay and atomic force microscopy (AFM) as well as the cytotoxicity, anticancer, and phytotoxicity of *C*. *excavata*.

**Method:**

Bacterial cell viability was performed by using microplate AlamarBlue assay. Atomic force microscopy was used to determine morphological changes in the surface of bacterial cells. Cytotoxicity and phytotoxicity were determined by brine shrimp lethality and *Lemna minor* bioassay. Caco-2 (colorectal adenocarcinoma) cell line was used for the evaluation of the anticancer effects.

**Result:**

Among the fractions tested, ethyl acetate (EA) fraction was found to be active with minimum inhibitory concentration (MIC) of 750 *μ*g/mL against *E*. *faecalis*, but other fractions were found to be insensitive to bacterial growth. Microscopically, the EA fraction-treated bacteria showed highly damaged cells with their cytoplasmic content scattered all over. The LC_50_ value of the EA fraction against brine shrimp was more than 1000 *μ*g/mL showing the nontoxic nature of this fraction. Chloroform (CH), EA, and methanol (MOH) fractions of *C*. *excavata* were highly herbicidal at the concentration of 1000 *μ*g/mL. EA inhibited Caco-2 cell line with an IC_50_ of 20 *μ*g/mL.

**Conclusions:**

This study is the first to reveal anti-*E*. *faecalis* property of EA fraction of *C*. *excavata* leaves, natural herbicidal, and anticancer agents thus highlight the potential compound present in its leaf which needs to be isolated and tested against multidrug-resistant *E*. *faecalis*.

## 1. Introduction

Since few decades, the emergence of antibiotic-resistant microbial infections has created alarming condition in global health care system due to significant increase in mortality in intensive care unit (ICU) [1, 2]. Development of resistant bacteria is governed by the indiscriminate use of the antibiotics. Other factors governing towards resistance include the usage of antibiotics as growth enhancers in livestock feed [3]. Hence, it is necessary to develop new antibacterial drugs to fight against multidrug-resistant pathogens. Medicinal plants have been subjected for treatment of many diseases from ancient times around the world. These products highly triggered the researchers to obtain natural and safe antibacterial agents due to lack of adverse side effects in comparison to the synthetic antibiotics, such as hypersensitivity, allergic reaction, and immunosuppression [4–10]. Atomic force microscopy (AFM) has been employed to scan the surface structures in nanoscales, and this property has been used to scan the morphology of bacterial cells [11–13].

*Clausena excavata* Burm. f. is a widespread plant in tropical and subtropical Asian regions. In popular medicine, leaves are used to treat wound, stomachache, headache, fever, malaria, snakebite, and poisoning [7, 11, 14]. *C*. *excavata* leaves contain high pharmacologically active coumarins such as furanocoumarins, xanthoxyletin, and nordentatin. These compounds are responsible for the antibacterial activities [11, 15–19]. In addition, our previous studies had reported that *C*. *excavata* leaves are rich in phenolic content quercetin, myricetin, and kaempferol, which help to suppress bacterial infections [20, 21].

Cytotoxicity testing provides important preliminary data for the selection of natural compounds with potential antimicrobial properties for future work [22]. Brine shrimp lethality bioassay plays an important role for getting information about the safety of compounds from crude extracts which exhibited antimicrobial activity. Due to the sensitivity of brine shrimp to a variety of chemical substances, many researchers had considered this assay for searching potent noncytotoxic natural compounds having biological and pharmacological properties [23–25]. Although previous studies showed antimicrobial activity of the bark, leaf, and stem of this plant [26, 27], there is no recorded data for anti-*Enterococcus faecalis*.

Use of herbicides has enhanced economic growth by increasing the production of food but its excessive use has resulted serious health implications to environment especially in soil and water ecosystem, thus caused health hazards to man and animals [28]. In comparison to synthetic compounds, natural compounds were found to be less toxic due to the absence of halogenated molecules, hydrophilic property, and a lower half-life, providing suppressive or inhibitory activity at lower concentrations [29]. The phytotoxicity (*Lemna minor*) assay is a general useful assay for screening plants having weedicidal property. Some natural antitumor compounds showed positive correlation with *Lemna* growth inhibition [30].

Cancer is one of the life threatening diseases responsible for high mortality all over the world. Colon cancer is the third leading cause of cancer-related deaths across the globe [31]. Recent studies done in Iraq showed an escalating rise in the incidence of colorectal cancer [32]. In some cases, even surgical or radio therapy could not cure or prevent the recurrence and metastasis of tumor. Hence, many investigators are focusing on natural products to have safe and effective anticancer drugs from natural source [33].

The antibacterial activity of *C*. *excavata* and other species of this genus *Clausena* had already been reported for their antibacterial property against some gram-positive and gram-negative bacteria [34–37], but no any records against *E*. *faecalis* have been reported during our literature survey, which are naturally present in gastrointestinal tract; therefore, it is recommended to traditional medicine practitioners to use this plant against gastrointestinal infections caused by this pathogen. Thus, this study was undertaken to evaluate the anti-*E*. *faecalis* as well as cytotoxic, phytotoxic, and antiproliferation activity towards Caco-2 cell line of *C*. *excavata* leaf solvent fractions.

## 2. Materials and Methods

### 2.1. Plant Material

The leaves *of C*. *excavata* plant were obtained from Pendang, Kedah, Malaysia (5°59′N, 100°28′E) on December 2010 and were identified by Dr. Shamsul Khamis (Resident Botanist) at the Biodiversity Unit, Institute of Bioscience, Universiti Putra Malaysia, voucher specimen (TI-013201-CE). Naturally grown plants in fields were collected. As it was not listed in endangered species, therefore, no specific permission was required to collect plant. Fresh leaves were dried in room temperature, powdered, and macerated in 1 : 5 dried leaf weights to solvent (petroleum ether (PET)) volume ratio for 3 days. The filtrate was collected, and the residues were subjected to further macerate with chloroform (CH), ethyl acetate (EA), and methanol (MOH) sequentially. The suspensions were collected, and solvents were removed under reduced pressure using rotary evaporator at 45-50°C to obtain crude extracts [19]. EA was sent for LCMS/MS analysis to screen for the presence of phytochemicals (Advance Chemistry Solution, GHOD Sdn Bhd ACD/Labs Inc., Malaysia).

### 2.2. Antibacterial Activity

The minimum inhibitory concentration (MIC) for the *E*. *faecalis* ATCC 49532 was determined by colorimetric indices in 96-well plates. The fractions were prepared using Mueller Hinton Broth (MHB) media to make concentration range (46.18-3000 *μ*g/mL) such that their total volume became 100 *μ*L in each well. Fully grown *E*. *faecalis* was diluted 1000 times in MHB media, and 100 *μ*L of this suspension was *aliquoted* to all wells containing 100 *μ*L of fraction such that bacteria will be approximately of 0.5 − 1.0 × 10^∧6^ CFU/mL. Untreated control was with only cells and media. All samples were in triplicates. The plate was sealed and incubated at 37°C for 18-20 h. The next day, all wells were visually checked to confirm the clear and turbid wells, and, then, 20 *μ*L of AlamarBlue dye was added, and the plate was incubated in the dark for 2 h in a shaking incubator at 37°C. Absorbance were recorded at 570 and 600 nm using a spectrophotometer (Thermo Scientific, USA), and the percent of inhibition of bacteria due to fractions was calculated by putting the absorbance values in the formula mentioned by Lancaster and Fields [38].

(1) $$\begin{array}{c} \text{Percent difference in reduction}=\frac{\left(\varepsilon OΧ\right)\lambda 2A\lambda 1-\left(\varepsilon OΧ\right)\lambda 1A\lambda 2\text{ of test agent dilution}}{\left(\varepsilon OΧ\right)\lambda 2A^{\circ }\lambda 1-\left(\varepsilon OΧ\right)\lambda 1A^{\circ }\lambda 2\text{ of untreated positive growth control}}\times 100, \end{array}$$

where *ε*OX is molar coefficient of AlamarBlue dye at different wavelengths *λ*1 (570 nm) and *λ*2 (600 nm) and *A* and *A*^o^ are absorbance of test and control wells, respectively.

### 2.3. Atomic Force Microscopy (AFM)

The EA fraction which was active against *E*. *faecalis* was subjected to the AFM study. Bacterial samples were prepared using inoculum of 2 − 3 × 10^7^ CFU/mL and treated with the MIC concentration of EA fraction while, untreated control contained only media and bacteria. After incubation for 3 h, cells were centrifuged at 5000 rpm for 5 min and washed twice with double distilled water (D.W). These cells were resuspended in 50 *μ*L of sterile distilled water, and 10 *μ*L of this suspension from treated and nontreated samples was spread in poly-l-lysine (0.01%) precoated silicon wafer slides and left it overnight for drying [11, 39, 40]. Sample scanning was carried out with AFM (Agilent Technologies-5500, AZ, USA) in tapping mode. All 2D and 3D topographical and pseudocolor images were analyzed using the PicoView 1.2 imaging analysis software.

### 2.4. Brine Shrimp (Artemia salina) Lethality Bioassay

A stock solution of artificial seawater was made by dissolving 38 g of sea salt in 1 L of distilled water; thus, filtered solution would be pH 7.4. This solution was kept in the hatching tray (22 × 32 cm) with perforated partition. On one half of this tray, 50 mg of brine shrimp eggs (San Francisco Bay Brand) was sprinkled and covered by aluminium foil on its top so that light could not penetrate inside, whereas lamp was kept on the other half so that larvae after growth will come out towards the light portion through partition pores. Stock solutions of fractions (10 mg/mL) were made in methanol, and 5, 50, and 500 *μ*L from this stock solution were dispensed in new vial in triplicates such that final concentration would be 10, 100, and 1000 *μ*g/mL, respectively. These vials were allowed for drying overnight. After 24 h of hatching, 10 active, motile nauplii were transferred in vial in triplicates by using a Pasteur pipette, and 5 mL of sea water was added in each vial and incubated for 24 h at 25-27°C under illumination. Vial with Etoposide as standard positive control at LD_50_ value of 7.5 *μ*g/mL was used. Only solvent was used as untreated control. Next day, the numbers of live and dead larvae was counted and find the percentage inhibition of larvae due to test fractions [7, 41, 42].

(2) $$\begin{array}{c} \%\text{inhibition}=100-\frac{\text{No}.\text{ of live larvae in test sample}}{\text{No}.\text{ of live larvae in untreated control}}*100. \end{array}$$

### 2.5. Phytotoxicity

A one-liter stock solution of inorganic E-medium was made in distilled water by mixing appropriate inorganic constituents [43], and pH 7 was maintained by adding potassium hydroxide pellets. Subsequently, a working solution was made by diluting the stock solution 10 times with distilled water. A stock solution (20 mg/mL) of different fractions of *C*. *excavata* leaves was made in methanol and dispensed 10, 100, and 1000 *μ*L of these solutions to the flasks in triplicates so that final concentration would become 10, 100, and 1000 *μ*g/mL, respectively, and left overnight for solvent evaporation. Next day, all flasks were filled with 20 mL of working E-medium solution and then 20 fronds of green, healthy *L*. *minor* were added to each flask and sealed the flask by parafilm and pricked the parafilm to make hole so that air passes inside the flask. In untreated control, there were no any compounds and served as the positive growth control, whereas standard plant growth inhibitor drug (paraquat at 0.015 *μ*g/mL) served as negative growth control. All these flasks were placed in growth cabinet for 7 days with maintained temperature at 27°C, relative humidity 55% ± 10%, and light intensity of 9000 lux. On the 7th day, the numbers of dead and live fronds in each flask was counted and calculated the percentage inhibition of each compound by using below mentioned formula [44].

(3) $$\begin{array}{c} \%\text{inhibition}=100-\frac{\text{Number of live fronds in test sample}}{\text{Total number of live fronds in untreated control}}\times 100. \end{array}$$

### 2.6. 3-(4,5-Dimethylthiazol-2-yl)-2,5,-diphenyltetrazolium Bromide (MTT) Assay of Fractions against Caco-2 Cell Line

The cytotoxicity of samples against human colon cancer Caco-2 (ATCC® HTB37™) cell line was determined by using the MTT assay [45, 46]. This cell line was obtained from the cell culture bank, Panjwani Centre for Molecular Medicine and Drug Research, ICCBS, Karachi. These cells were maintained in Dulbecco's Modified Eagle Medium (DMEM) supplemented with 10% fetal bovine serum and 1% nonessential amino acid and incubated at 37°C in 5% CO_2_ incubator. Fetal bovine serum is required for proper growth, and it is also required to neutralize trypsin during trypsinization process. DMEM was replaced every 48-72 h until 80% confluence was achieved.

Cells at passage number 5 were seeded into 96-well plates at density 5000 cells/well in 100 *μ*L culture medium. After 24 h incubation, the medium was replaced with fresh medium containing different fractions (EA, CH, and MOH) in various concentrations (100, 50, 25, and 12.5 *μ*g/mL) and incubated for 24 h. Untreated well served as positive growth control. After incubation, old medium was replaced by fresh medium containing 0.5 mg/mL of MTT dye and incubated further for 4 h. The medium containing MTT dye was removed, and 100 *μ*L of dimethyl sulfoxide (DMSO) was added to all wells to solubilize the formed formazan crystals. After 10 min, plate was shaken for 30 sec and absorbance was recorded at 570 nm in spectrophotometer (Multiskan GO, Thermo Scientific). In this assay, doxorubicin at 0.501 *μ*g/mL was used as a standard drug. The percentage of cytotoxicity of compounds was determined by comparing with the untreated positive growth control. All experiments were performed in triplicate [45].

(4) $$\begin{array}{c} \%\text{cytotoxicity}/\text{inhibition}=100-\frac{\text{O}.\text{D of treated well}-\text{O}.\text{D of media control}}{\text{O}.\text{D of untreated control}-\text{O}.\text{D of media control}}\times 100. \end{array}$$

## 3. Statistical Analysis

Data of each fraction were presented in IC_50_ (mean ± S.D) values and compared with IC_50_ value of positive drug control. One-way analysis of variance (ANOVA) was used in the GraphPad Prism software version 5.0 and analyzed with level of significance (*P* < 0.05).

### 3.1. Results

### 3.2. Antibacterial Study

The change in color of the dye from blue to pink indicated the reduction of dye due to reducing environment of viable cells, whereas blue color indicated nonviable cells. The decrease in AlamarBlue dye reduction suggested that the fractions have antibacterial properties. The antienterococcal activities of different fractions of *C*. *excavata* leaves are presented in Table 1. The study showed that the EA fraction was found to be active against *E*. *faecalis* at MIC of 750 *μ*g/mL, whereas other fractions were found to be inactive. Thus, EA fraction was carried out for the AFM study.

### 3.3. Atomic Force Microscopy

**Figure 1:**
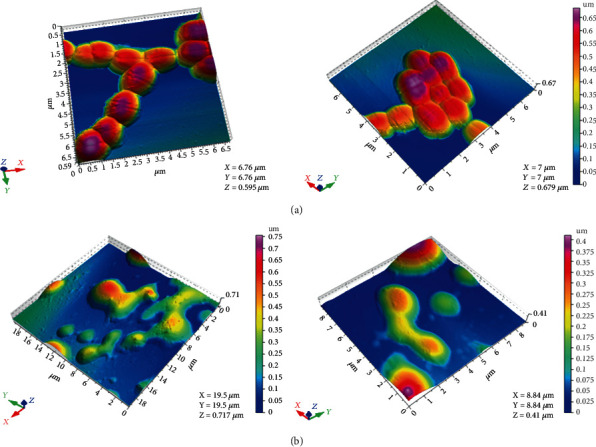
Atomic force microscopy images of *E*. *faecalis* (ATCC 49532): (a) untreated control and (b) ethyl acetate- (EA-) treated cells at 750 *μ*g/mL. Different colors are according to the height of the image. Pink color shows the highest height, and color changes to red, orange, yellow, green, and blue with decreasing height.

AFM technique was used to visualize the morphological changes on bacterial strain due to antibacterial effect of ethyl acetate fraction at its MIC value. The images of untreated *E*. *faecalis* cells showed cocci arranged in clusters, with some short chains. These cells were typically oval shaped, smooth surfaces with a mean diameter of 0.5-1.0 *μ*m (Figure 1(a)). No visible pores or rupture were seen on surface indicating the well-preserved structural integrity. Upon treatment with EA fraction, significant morphological damages were seen with loss of cluster formation (Figure 1(b)). Large amount of cytoplasmic content was spilled all over, thus confirming the antibacterial effect of EA fraction.

### 3.4. Brine Shrimp Lethality Assay

**Figure 2:**
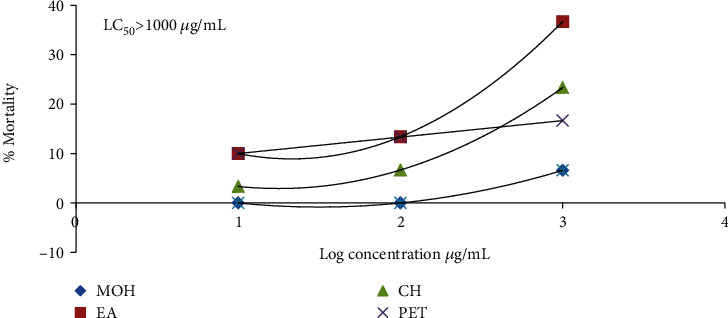
Brine shrimp (*Artemia salina*) lethality bioassay of different fractions of *C*. *excavata* leaves. EA at 1000 *μ*g/mL showed significant (*P* < 0.05) difference as compared to other fractions.

Among different fractions, only ethyl acetate showed slight toxicity (36.6% lethality) against *A*. *salina* larvae at 1000 *μ*g/mL whereas lethality of other fractions even at 1000 *μ*g/mL was less than 25% showing the nontoxic nature of these fractions (Figure 2). This result showed that these fractions are noncytotoxic even at higher concentrations so these could be used in ethnomedicine with minimal side effects.

### 3.5. Phytotoxic Activity

EA, CH, and MOH fractions were found to be highly active at 1000 *μ*g/mL, but inactive in lower concentrations of 100 and 10 *μ*g/mL, while PET showed moderate phytotoxic activities at same dose (Table 2). The IC_50_ of paraquat was 0.02 *μ*g/mL which was much lower as compared to EA fraction. There was significant difference (*P* < 0.001) between fraction and drug control IC_50_ value.

### 3.6. Effect of C. excavata Leaf Fractions on Caco-2 Colorectal Cancer Cell Lines

The two fractions (CH and EA) of *C*. *excavata* showed *in vitro* growth inhibition effects on the Caco-2 cell line. At 100 and 50 *μ*g/mL, EA fraction showed more than 90% inhibition, whereas CH fraction showed 70-80% inhibition at 50 *μ*g/mL. IC_50_ of EA and CH fraction were found to be 20 and 40 *μ*g/mL, respectively, which were significantly higher (*P* < 0.001) as compared to doxorubicin (IC_50_ = 0.5 *μ*g/mL). Therefore, EA fraction found to be more suitable for further study on colorectal cancer activity. The MOH showed less effect throughout the range of tested concentrations in Caco-2 cell line (Table 3).

## 4. Discussion

With the escalation of multidrug-resistant bacterial pathogens, the advancement of ethnopharmacology became a focus area for researchers in the discovery of alternative natural drug with potent antimicrobial activities [47]. *C*. *excavata* leaves have been widely used in folklore medicine, but only a few scientific evidences proved its therapeutic properties and mechanism of action [14]. *C*. *excavata* was reported to possess antibacterial activity against *Bacillus* subtilis, *Micrococcus luteus*, *Staphylococcus aureus*, *Escherichia coli*, *Klebsiella pneumonia*, *Proteus vulgaris*, and *Shigella flexnari* [11, 48]. In this study, the *C. excavata* leaves fractions were first time reported for their anti-*E*. *faecalis* properties by using AlamarBlue assay. In this assay, resazurin, a redox-sensitive dye which is originally blue in color has the ability to penetrate the viable cells. Inside the cell, due to reducing environment, resazurin is reduced and converted to resorufin giving pink coloration [49]. Thus, changing of color from blue to pink shows the viability of cells. This assay is rapid, simple, and low cost with inexpensive instrument; therefore, it is widely used in several viability/proliferation assays for bacteria, fungi, cancer lines, and other viable cells. By using this assay, we assigned the MIC value to that concentration in which there was blue coloration, and below this concentration, there was pink coloration due to growth of bacteria. Among different fractions, EA fraction showed good anti-*E*. *faecalis* activity and its further analysis by AFM technique showed scattered cytoplasmic content confirming their lost cellular integrity.

The nonpolar solvent (EA) fraction was active at 750 *μ*g/mL; hence, it proved that the nonpolar active compounds present in nonpolar fraction are responsible for the anti-*E*. *faecalis* activity, as previously reported in other plants [50]. Coumarin and carbazole derivatives, isolated from nonpolar solvent extract of roots and leaves of *C*. *excavata*, have been reported for their antimicrobial, wound healing, and antioxidant properties [15–18]. Previous studies reported that the mechanism of action of natural drug was focused to show their effects on surface morphology [51].

In brine shrimp lethality assay, the nonpolar solvent (EA and CH) fractions reduced brine shrimp survival as compared with the other fractions at 1000 *μ*g/mL. This may be due to high coumarin content in these fractions [25]. Earlier reports have shown that substances from natural products were known as toxic if the LC_50_ ≤ 1000 *μ*g/mL. Thus, EA and CH fractions were considered nontoxic on brine shrimp since the LC_50_ is more than 1000 *μ*g/mL [25, 52]. The outcome is in agreement with article reported by Albaayit et al. [15], who demonstrated that EA and CH fractions did not inhibit Vero, a kidney epithelial cell line.

Since last few decades, overuse of herbicides led to the emergence of herbicide-resistant weeds, because of which conventional synthetic herbicides became less effective and caused low crop yield with massive economic loss and health and environmental-related concerns. Therefore, herbicide researchers are trying to make new herbicides by isolating potent compounds from natural sources [53]. The CH, EA, and MOH fractions showed the most significant phytotoxic (100% mortality) effect at tested concentrations of 1000 *μ*g/mL, while PET showed moderate phytotoxic activities at same dose. The findings are similar to that of previous studies, which have reported that plants containing phenolic compounds and their derivatives as potential inhibitors of seedling growth [54, 55].

Till date, many anticancer compounds have been isolated from this plant. Excavatine A, a carbazole alkaloid, has been reported for its anticancer effect on adenocarcinomic human alveolar basal epithelial cells and cervical cancer. Clausine-TY, a carbazole alkaloid, showed anticancer activity against CEM-SS cell line [56]. Propyl-cannabinol allyldimethylsilyl ether compound showed anticancer effect on HT29, MCF-7, and HepG2 cell lines [57]. Clausine B, a carbazole alkaloid, showed antiproliferative activities against MDA-MB-231, HeLa, CAOV3, and HepG2 cell lines [58]. Three carbazole alkaloids (Clausine-E, Murrayanine, and Clauszoline J) also showed potent antiproliferative activity against NCI-H187, MCF-7, and KB cell lines [59]. The inhibition of Caco-2 cell line growth by *C*. *excavata* (EA and CH fractions) might be due to the presences of phenolic compounds like coumarin that induces apoptosis by activating the caspase-3-dependent apoptotic pathway and the mitochondrial pathway, by downregulating antiapoptotic genes (Bcl-2 and Bcl-xL), upregulating caspase-3 with the release of cytochrome c. and arrest and premature aging, and enhancing the immune system to destroy cancer cells [60–62].

## 5. Conclusion

This study first time reports the anti-*E*. *faecalis* activity of ethyl acetate fraction of *C*. *excavata* leaves; thus, the antibacterial compounds previously isolated from this plant may be active against MDR *E*. *faecalis* and could be used in gastrointestinal infections after further investigation. The ethyl acetate fraction is noncytotoxic to brine shrimp larvae and possesses anticolorectal activity. Due to its phytotoxic property, it could be used as a herbicide. In our future work, we will test its *in vivo* efficacy in the treatment of gastrointestinal infections caused by *E*. *faecalis.*

## Figures and Tables

**Table 1:** AlamarBlue assay (% inhibition) and minimum inhibitory concentration (MIC) (*μ*g/mL) of different fractions of *C*. *excavata* leaves against *E*. *faecalis.*

Fraction names	*Enterococcus faecalis* (ATCC 49532)	
	MIC (*μ*g/mL)	% inhibition (mean ± S.D)
Chloroform (CH) fraction		Not active
Ethyl acetate (EA) fraction	750	80 ± 2.5
Methanol (MOH) fraction		Not active
Petroleum ether (PET) fraction		Not active

**Table 2:** *In vitro* phytotoxicity bioassay of different fractions of *C*. *excavata* leaves against *L*. *minor.*

Concentration (*μ*g/mL)	% inhibition at different concentrations				
	Methanol (MOH)	Ethyl acetate (EA)	Chloroform (CH)	Petroleum ether (PET)	Paraquat
1000	100	100	100	48	
100	0	25.7	8.57	14.28	
10	0	0	0	5.7	
IC_50_ value (*μ*g/mL)	313.1	213.3	283.1	>1000	0.02

**Table 3:** MTT assay (% inhibition) of different fractions of *C*. *excavata* leaves against Caco-2 cell line.

Concentration (*μ*g/mL)	Ethyl acetate (EA)	Chloroform (CH)	Methanol (MOH)	Doxorubicin
100	97.7	97.003	<20	
50	96.2	74.1	<20	
25	88.39	24.2	<20	
12.5	37.41	21.1	<20	
IC_50_ value (*μ*g/mL)	20	40	Not active	0.501

## Data Availability

Data used to support the findings of this study are included within the article
